# Effectiveness of Synchronous Telerehabilitation Versus Face-to-Face Physical Therapy in Older Adults Who Are Frail: Protocol for a Randomized Controlled Trial

**DOI:** 10.2196/72318

**Published:** 2025-09-16

**Authors:** Igor Cigarroa, Daniel Reyes-Molina, Felipe Vargas-Rios, Gustavo López-Alarcón, Sergio Jara-Aceituno, Cristobal Riquelme-Hernández, Rafael Zapata-Lamana, María Antonia Parra-Rizo

**Affiliations:** 1 Escuela de Kinesiología Facultad de Ciencias de la Salud Universidad Católica Silva Henríquez Santiago Chile; 2 Escuela de Kinesiología Facultad de Salud Universidad Santo Tomás Santiago Chile; 3 Escuela de Kinesiología Facultad de Ciencias de la Salud Universidad Arturo Prat Victoria Chile; 4 Escuela de Educación Universidad de Concepción Los Ángeles Chile; 5 Faculty of Health Sciences Valencian International University Valencia Spain; 6 Department of Health Psychology Faculty of Social and Health Sciences Universitat de Miguel Hernández d'Elx Elche Spain

**Keywords:** older person, fragility, telerehabilitation, physical fitness, functional status, quality of life

## Abstract

**Background:**

Older adults who are frail face significant barriers to physical activity, and innovative solutions such as synchronous telerehabilitation (STR) may provide a viable alternative to traditional face-to-face programs. However, less is known about its effectiveness compared to a face-to-face program.

**Objective:**

This study aims to compare the effectiveness of an STR program versus a face-to-face physical therapy (FPT) program in the primary outcomes (lower body strength and cardiorespiratory fitness) and secondary outcomes (upper limb strength, dynamic balance, static balance, number of steps, functional status, and quality of life) in older adults who are frail.

**Methods:**

In a randomized, blinded, parallel-group controlled trial, all older adults who are frail and aged ≥60 years of both sexes from the Los Angeles Comprehensive Center for Older Adults will be invited to participate. A total of 58 older adults who are frail (aged ≥60 years) meeting the eligibility criteria will be randomly assigned to either an STR group (n=29, 50%) or an FPT group (n=29, 50%). Participants will engage in 1-hour multicomponent exercise sessions twice weekly for 12 weeks. The telerehabilitation group will participate via videoconferencing, supervised remotely by technical staff, while the face-to-face group will attend in-person sessions. Outcomes will include lower limb strength (sit-to-stand test), cardiorespiratory fitness (2-minute step test), upper limb strength (grip strength test), balance (Timed Up and Go, and single-leg stance), quality of life (36-item brief health survey), functional status (functional independence measure), and daily steps (pedometer).

**Results:**

Recruitment will be conducted from April to May 2025. The intervention will run from June to August 2025, follow-up assessments will be completed by September 2025, and data analysis will be finalized by December 2025. Manuscript drafting and submission are planned for March 2026. We hypothesize that STR will yield equal or superior improvements in both primary and secondary outcomes compared to FPT in older adults who are frail.

**Conclusions:**

STR is emerging as a viable and effective alternative to in-person physical therapy for older adults with frailty. This approach has the potential to improve physical condition and functional and quality of life indicators, overcoming geographical and logistical barriers and optimizing therapeutic resources.

**Trial Registration:**

ClinicalTrials.gov NCT06784245; https://clinicaltrials.gov/study/NCT06784245

**International Registered Report Identifier (IRRID):**

PRR1-10.2196/72318

## Introduction

### Background

Worldwide, the prevalence of frailty in people aged ≥60 years ranges from 7% to 24% [1]. In Latin America and the Caribbean, the prevalence of frailty ranges from 7% to 42.6% [2]. A recent study conducted in Chile found that 10.9% of individuals aged >60 years are frail, including 7.7% of men and 14.1% of women affected. The study also reported that the prevalence of frailty in Chile increases significantly with age, reaching 58% for men and 62% for women after the age of 80 years [3]. Frailty is a clinical stage with a multifactorial etiology that involves disorders of multiple interconnected physiological systems [4]. Its diagnosis is commonly based on 5 components: muscle strength, gait, exhaustion, weight loss, and chronic fatigue [5]. People who are frail have a higher risk of falls, disability, long-term care, and death [6]. Therefore, prevention and early detection have become significant challenges for public health and clinical practice [7].

The first-line therapy for managing frailty should include a multicomponent physical activity program with a resistance-based training component [8]. Recent evidence shows the efficacy of a multicomponent exercise program as a strategy to prevent [9] and reverse frailty to prefrailty. In addition, it has been seen that a multicomponent exercise program can improve physical fitness, particularly resistance and balance [10,11], and quality of life [12] in older adults with this clinical stage. Despite the many benefits of exercise, participation is low among older adults, with relatively few older adults who are frail meeting the recommendations for exercise [13,14].

In Chile, the “More Autovalent Older Adults” (*Más Adulto Mayor Autovalente)* program, implemented by the Ministry of Health, is one of the primary national strategies aimed at promoting and maintaining functionality and physical fitness in older adults [15]. International and national experience indicates that most of these programs are group-based aerobic exercises and the promotion of self-care. Furthermore, participation rates may fluctuate due to factors such as time, available space, weather conditions [16,17], or distance between home and the exercise location, representing a barrier for older adults to join or maintain their attendance at out-of-home programs [18]. As a result, older adults are more likely to develop sarcopenia or frailty due to the decrease in physical activity [19]. In addition, exercise instructors have a more difficult time offering individual instruction to their older adult participants when the physical activity program is carried out with large groups and heterogeneous health characteristics and may have trouble providing effective feedback relevant to their cognitive characteristics [19]. Home-based exercise alternatives have been proposed to overcome the limitations of conventional programs, such as videos, online platforms, and mobile apps [20]. However, this modality does not offer direct feedback, making it difficult for older adults to benefit fully, and the risk of injury is higher [19].

In this context, telerehabilitation has been proposed as a new intervention method to prevent or improve frailty. Telerehabilitation refers to the delivery of rehabilitation services to patients remotely, using information and communication technologies [21]. Communication between the patient and the rehabilitation professional may occur through a variety of technologies, such as the telephone and internet‐based videoconferencing [22]. Widely accepted advantages exist for the use of telerehabilitation rather than the more traditional, clinic-based service delivery model. Telerehabilitation facilitates access to rehabilitation opportunities [23], reduces health care disparities, and allows patients to receive services in the comfort and privacy of their own homes [24,25]. This model eliminates geographical barriers [26-28] and reduces both the time and out-of-pocket expenses associated with transportation. In addition, it diminishes the reliance on others for mobility or the need for adapted transportation for some older individuals who are frail [24,27,29,30]. Consequently, telerehabilitation is associated with increased session attendance and functional improvements comparable to or even superior to those achieved with in-person care [31].

Synchronous telerehabilitation (STR) involves real-time, two-way video and audio communication between the exercise instructor and participant, allowing for supervised training using high-speed telecommunications technologies. This would allow for real-time interactions between exercise instructors and older adults living in their homes [32,33]. Three international studies have evaluated the effectiveness of STR programs for older adults at high risk of falls and sarcopenia. Significant improvements were found in physical condition (leg strength measured with the chair stand test), static balance (Berg Balance Scale), and decreased sarcopenia [19,33,34]. Telerehabilitation is considered a viable alternative to traditional rehabilitation, as it reduces the use of outpatient resources and improves quality of life. However, further randomized controlled trials are required to evaluate its efficacy in older adults with chronic diseases, frailty, and sarcopenia [35-37].

To date, few studies in Chile have reported using these mobile technologies (asynchronous telerehabilitation) to support the treatment of older adults on physical fitness and functional status [38]. Nonetheless, no studies have been found examining the effects of an STR program on the physical capacity, functional status, and quality of life of older people who are frail. Few studies have rigorously examined whether patients achieve comparable benefits from in-clinic versus remote physiotherapy telerehabilitation sessions. Much of the existing research implements a hybrid model of combined home and clinic sessions rather than just remote delivery [39]. This is because the combined service delivery increases confidence in the accuracy of diagnosis and assessment of progress by providing regular opportunities for physical contact and manipulation of a patient’s extremities and joints. Regardless of whether a combined or remote-only model is used, studies comparing telerehabilitation with clinic-based physiotherapy consistently suggest that both approaches yield comparable results [39,40].

### Objectives

In this context, this study aims to analyze whether the effectiveness of an STR program is better than a face-to-face physical therapy (FPT) program in the primary outcomes (lower body strength and cardiorespiratory fitness) and secondary outcomes (upper limb strength, dynamic balance, static balance, number of steps, functional status, and quality of life) in older adults who are frail. We hypothesize that (1) both STR and FPT programs improve primary (lower body strength and cardiorespiratory fitness) and secondary outcomes (upper limb strength, dynamic balance, static balance, number of steps, functional status, and quality of life) in older adults who are frail and (2) STR produces equal or greater improvements compared to face-to-face programs in these outcomes.

## Methods

### Study Design

This study is designed as a blinded (assessor and statistician), parallel-group, randomized controlled trial. The CONSORT (Consolidated Standards of Reporting Trials) guidelines for clinical trials will be followed [41,42], with reporting on social and psychological interventions [43], and the SPIRIT (Standard Protocol Items: Recommendations for Interventional Trials) guidelines for protocol studies (Multimedia Appendix 1) [44]. The clinical trial was registered on ClinicalTrials.gov (NCT06784245).

### Study Setting and Participants

The Centro Integral del Adulto Mayor (CIAM; Comprehensive Center for Older Adults) in the city of Los Angeles, Biobío region, Chile, will be invited to participate. CIAMs aim to provide care, promotion, and support to individuals aged ≥60 years. These centers are managed by local municipalities and serve as spaces for social interaction and activities, including workshops, meetings, and medical and social care tailored to older adults. CIAM Los Angeles was selected due to its central role as the primary location where older adults from throughout the city gather. This provides access to a diverse group of older adults, including those who are frail and meet the study’s selection criteria. A collaboration agreement was created with CIAM to be able to carry out outcome measurements and the FPT program. In addition, CIAM has a space equipped with a 50-inch smart television and physical activity equipment so that a health professional can guide telerehabilitation sessions.

### Power Calculation

**Figure 1:**
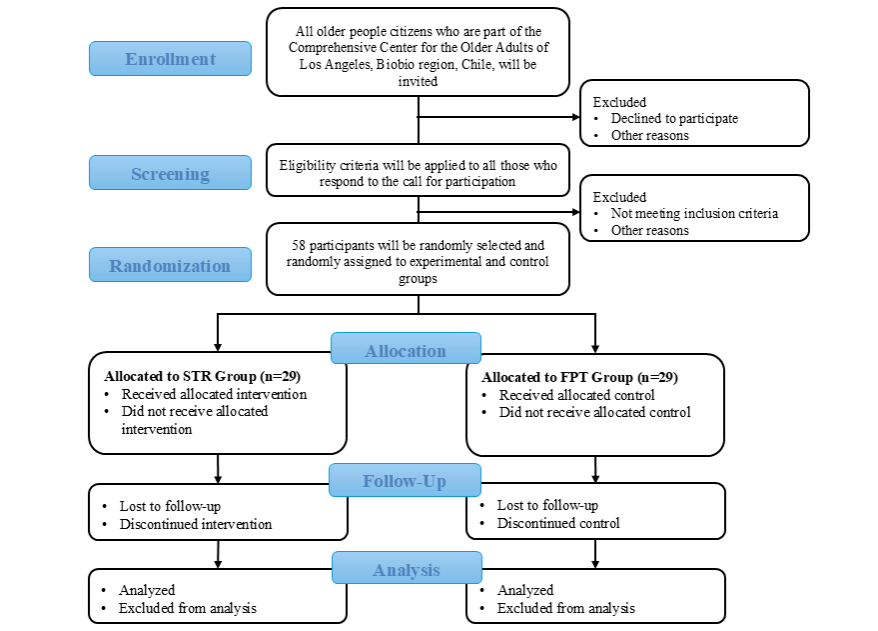
Flowchart of the synchronous telerehabilitation (STR) intervention [41]. FPT: face-to-face physical therapy.

The sample size was calculated a priori using G*Power 3.1.9.7. A total of 52 participants in total are required (26 in each group), considering Cronbach α=.05, 1-β=.8, and effect size=0.8 (lower limb strength based on the results of a previous study) [19]. Considering the possibility of abandonment, 6 participants were added (N=58; n=29 in each group; Figure 1 [42]).

### Recruitment of Participants

All individuals aged ≥60 years belonging to CIAM Los Angeles will be invited to participate. Research support staff will conduct recruitment in person or by telephone. To be eligible for participation, individuals must meet the following inclusion criteria: (1) both sexes aged ≥60 years, (2) older adults from the CIAM, (3) older adults diagnosed with frailty according to the modified Fried phenotype scale [5], (4) ability to stand up and walk ≥10 m without assistive devices, (5) Abbreviated Mini-Mental State Examination >13 points [45], and (6) no medical contraindication for physical exercise. In addition, older adults must have a smartphone or tablet and an internet connection at home. Computer or internet literacy is not required as an eligibility criterion. Exclusion criteria include older adults with fractures, recent acute myocardial infarction, associated cardiovascular pathology, severe acute respiratory failure, high blood pressure, uncontrolled diabetes mellitus, limitations following instructions, as well as individuals participating in another exercise program during the project (Figure 1).

### Randomization

The sample will be selected by quota until a size of 58 participants is reached. The selection will be random among those who meet the inclusion and exclusion criteria. Participants will be randomized by research staff using a computer-generated sequence and concealed allocation to one of the following two groups: (1) the STR group, which will receive STR, or (2) the FPT group, which will receive FPT. A simple randomization computational sequence stratified by gender, age, and frailty will be used to obtain a balance between men and women, age ranges, and severity of frailty in both groups. The randomization sequence will be concealed using sequentially numbered, opaque, sealed envelopes to ensure allocation concealment (Figure 1).

### Procedure

**Figure 2:**
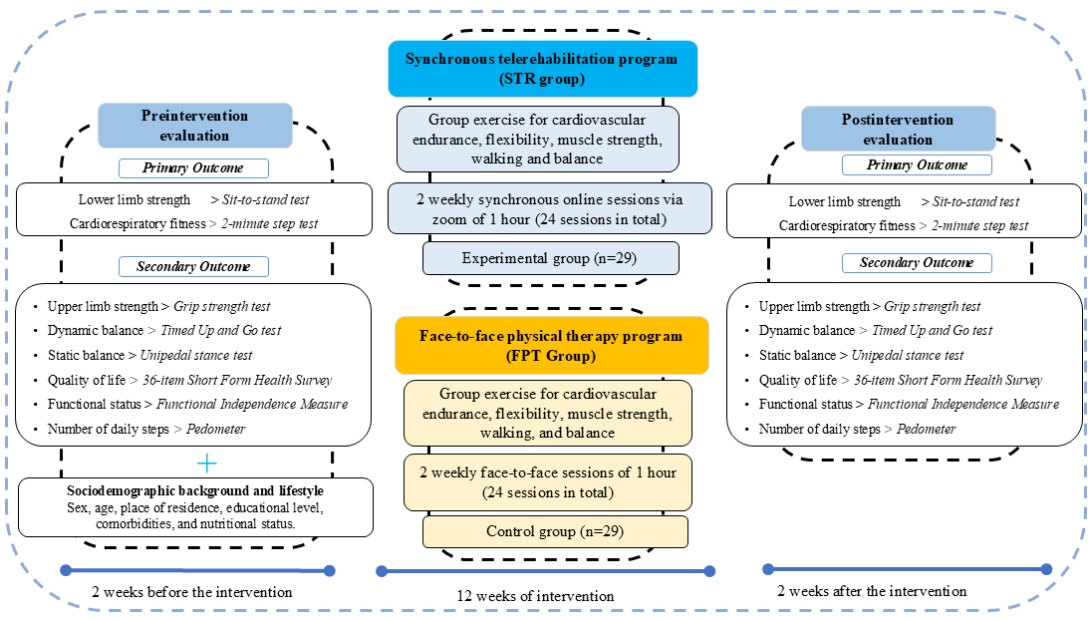
Study design of the synchronous telerehabilitation intervention program.

Primary outcomes (lower body strength and cardiorespiratory fitness) and secondary outcomes (upper limb strength, dynamic balance, static balance, number of steps, functional status, and quality of life) will be assessed face-to-face 2 weeks before the initiation of the intervention in both groups (Figure 2).

In addition, at that time, STR group participants will be provided with a tablet and home exercise equipment (exercise mats, resistance bands, cones, and dumbbells). If exercise materials are lost or damaged, they will be replaced. A home visit will be conducted to identify barriers to exercise and assess the most suitable location to carry out the STR sessions. One week before the intervention, there will be 2 induction sessions on the proper use of sports equipment, as well as video calls. Written and verbal instructions will be provided outlining the simple steps necessary to connect to an online meeting and facilitate participation in the STR program, as well as the proper use of the home exercise equipment. In addition to the induction sessions, asynchronous material was provided to address questions related to connectivity and the use of devices such as tablets and watches.

At the beginning of the interventions, all participants will be given a hydration kit (cotton towel and a water bottle) and a smartwatch (Fitbit Inspire 2, Google LLC) to measure continuous heart rate monitoring during exercise sessions to maintain exercise intensity, and pedometer (OMRON HJ-321, Kyoto, Japan) to record the number of daily steps each participant takes. (All the equipment that will be delivered to participants is guaranteed).

During the intervention period, nutrition guidance and exercise education will be provided to all participants once every 4 weeks. Throughout the 12-week intervention period, the participants will be encouraged to maintain the same physical activity levels and calorie intake as before participating in the study. During the education sessions, we will inform the participants to inform the support personnel holding the education sessions about any changes in physical activity levels or nutrition intake. A record will be kept of the number of sessions completed by each participant, which will serve as an indicator of adherence to the program. In addition to session attendance, retention rates (including reasons for nonattendance or withdrawal) and interactions with the technical support team will be recorded.

A postintervention assessment will be conducted after the last week of the 12-week program (Figure 2).

All data collection will be carried out in a face-to-face meeting with older adults at a care center. Technical staff will be trained to ensure an unbiased procedure. Measurements will be taken individually in a place with optimal conditions of privacy, temperature, and humidity. Evaluations will be conducted by health care professionals. The person evaluating will be different from the one performing the interventions.

### Interventions

#### Overview

Regardless of the group (STR or FPT), each session will consist of a warm-up activity (5-10 minutes), main exercise activity (30-40 minutes), and cool-down activity (5-10 minutes) following the guidelines of a multicomponent exercise program, Vivifrail [46]. Exercise intensity will be controlled based on the Rating of Perceived Exertion (RPE) [47] on the Borg scale [48] and heart rate measured with a smartwatch [49] before, during, and after each exercise session according to the guidelines of the American College of Sports Medicine [47]. The warm-up and cool-down activities will include stretching and walking in place (9≤RPE≤10 and 40<55% maximum heart rate (HRmax), while the main exercise activity will consist of strength, resistance exercise, cardiovascular exercises, and balance exercises (to prevent falls) performed using dumbbells, color-coded resistance bands (Thera-Band; Hygienic Corp) [50] and a chair (11<RPE≤15 and 55<70 HRmax).

To ensure safety and compliance, exercise training will be supervised by a health professional (physical therapist) who has been appropriately trained (research support staff). This health professional will provide one-on-one instructions to each participant according to the target RPE and HRmax (measured with a smartwatch) for each session. All participants will interact with the same health professionals.

Specifically, in each session, the resistance exercise will be carried out through squats, dumbbells, or chair-based exercises using a color band (as an example of the exercise progression: yellow [level 1] band during weeks 1-4, red [level 2] band during weeks 5-8, and green [level 3] band during weeks 9-12). This routine will focus on the main muscle groups in the shoulders, arms, thighs, hips, and calves, including 3 or 4 sets with 8 to 15 repetitions per set [51]. The balance exercise will include 2-legged standing, tandem standing, single-legged standing, semitandem standing, tandem walking, turning in a circle around the chair, and exercises such as toe-standing exercises, which will focus on postural muscle groups. Cardiovascular exercise will consist of walking at a pace at which you can maintain a continuous conversation, but it costs a little effort. Walking duration may increase (8-15 minutes) as the sessions go by. The total exercise duration progressively increased from 40 to 60 minutes over the course of the intervention period. Previous international studies that analyze STR programs have used similar exercise protocols [19,52].

To increase adherence to the interventions and avoid eliminating participants, WhatsApp groups will be created, and messages will be sent to remind participants to participate in the exercise sessions.

#### STR Program (STR Group)

During the exercise session, the participants turn on the tablet PC, watch the instructor perform the exercise, and follow the instructor’s movements. For the instructor to observe the correct movements of the participants, the resistance and balance exercises are performed in the frontal and sagittal planes. During each session, a technical staff member will supervise up to 4 or 5 participants who are exercising remotely at home using a real-time videoconferencing app (Zoom Communications). The videoconference session will be shown on a television screen located in an older adult care center. Once the technical staff has been assigned to participants, their supervision will not be interchangeable. Participants will be able to see and interact with both the health care professionals and other participants. The older adults, from their homes, will complete 1-hour sessions twice a week for 12 weeks (Figure 2).

#### FPT Program (FPT Group)

The exercise sessions will take place in a center for older adults, lasting 40 to 60 minutes, twice a week for 12 weeks, and will include a combination of cardiovascular, balance, strength, flexibility, and gait exercises (Figure 2).

### Outcome Measures

#### Overview

Physical fitness, functional status, and quality of life will be examined before and after the interventions. All measurements will be performed by a health professional (technical staff will be trained), blinded to the group allocation scheme, which will guarantee an unbiased procedure. In addition, all measurements will be conducted in a face-to-face setting and obtained individually in an environment with optimal conditions of privacy, temperature, and humidity, such as an older care center collaborating with the project. Data will be recorded on standardized forms and entered into a secure access database that contains quality control checks (eg, range checks and notifications for missing data). The main outcome for this trial will be lower body strength (assessed in the 30-second chair stand test) and cardiorespiratory fitness (evaluated using the 2-minute step test). Secondary outcomes included upper limb strength, dynamic balance, static balance, number of daily steps, functional status, and quality of life.

#### Primary Outcomes

##### Lower Limb Strength

This will be measured using a 5-repetition sit-to-stand test. The older adults will be asked to stand up and sit down, as quickly as possible, 5 times from a chair without armrests located against a wall. The time will be measured from the beginning of the movement until the older adult manages to stand up for the fifth repetition. The older adult’s arms should be crossed over the chest during the test. The time will be recorded in seconds and tenths of a second [53,54].

##### Cardiorespiratory Fitness

This will be measured by a 2-minute step test. The evaluator will count the number of full steps completed by the participant in 2 minutes. A full step is defined as a step performed while raising the knee up to a height corresponding to the midpoint between the patella and the iliac crest [54].

#### Secondary Outcomes

##### Upper Limb Strength

This will be estimated using the grip strength test, which uses a previously calibrated Baseline hydraulic dynamometer. This evaluation will be carried out with the older adult seated on a chair with a backrest, shoulders adducted, elbow flexed at 90°, forearm, and wrist in a neutral position. The evaluated arm should not rest on any surface, and the dynamometer should be used vertically. Older adults will be asked to perform a maximum grip force with their dominant hand for 3 seconds, resting for 1 minute between each repetition, making 2 attempts. The highest grip value of the repetitions will be used. This protocol is described in Encuesta nacional de salud 2016 to 2017.

##### Dynamic Balance

This will be measured with the Timed Up and Go test. The older adult will be seated on a chair without armrests, with the back against the backrest and feet touching the ground. The older adult will be asked to stand up and walk as usual to a cone located 3 m away, turn around, and return to sit down. This test controls the time it takes to complete the circuit, starting from the moment the older adult lifts their back from the chair and ending when returning to the start position. The scale is normal ≤10 seconds, with a slight risk of falling at 11 to 20 seconds and a high risk of >20 seconds [55].

##### Static Balance

This will be measured using the single-leg stance test. The participants will be asked to cross their arms over their chest, rest their hands on their shoulders, and triple-flex one leg at 90°, staying as long as possible on 1 foot (maximum of 30 s). The test will be repeated 3 times, and the best time obtained will be considered for the study. According to national studies, it is assumed that an older adult has a high risk of falling due to not being able to maintain this position for ≥5 seconds [55].

##### Quality of Life

This will use the 36-item Short Form Health Survey (SF-36) questionnaire [56,57] in its Spanish version [58]. The SF-36 is a self-report instrument that contains 36 questions from 8 dimensions related to people’s health: physical function, physical role, bodily pain, vitality, social function, emotional role, mental health, and general health [59]. The score obtained corresponds to values on a scale of 0 to 100, where higher scores indicate better health [59]. The SF-36 is adapted and validated in Chilean older adults, presenting adequate indicators of general Cronbach α internal consistency of .88 [60].

##### Functional Status

It will be measured by using the Functional Independence Measure. It is composed of 18 items, with a total score ranging from 18 to 126, and it allows quantifying the demand for help from third parties that a person needs to perform their daily life activities. The evaluated items include self-care activities, sphincter control, locomotion, mobility or transfer, and social cognition. For each evaluated activity, the score ranges from 1 (totally dependent) to 7 (totally independent) [61]. The Functional Independence Measure is one of the most widely used instruments to carry out functional evaluation in rehabilitation, validated for the older adults [62], presents clinical validity, agreement between evaluators, test-retest, and intraobserver reliability, and has been widely used in Chile [63].

##### Number of Daily Steps

Each participant will wear a digital pedometer for 7 consecutive days. The total number of steps recorded during this period will be summed and divided by the number of days the pedometer was worn to calculate the average daily step count. Each participant will receive instructions and an illustrative guide with specific care of the equipment. We will use the classification proposed by Tudor-Locke and Bassett, which indicates that the number of steps represents the level of physical activity: <2500 steps/day (basal activity); 2500 to 4999 steps/day (limited activity); 5000 to 7499 steps/day (low activity); 7500 to 9999 steps/day (mildly active); 10,000 to 12,499 steps/day (active); >12,500 steps/day (highly active) [64].

#### Sociodemographic Background

The sociodemographic characteristics of the participants will be recorded, including age, gender (male or female), geographical origin (rural or urban), educational level (basic education: <8 years, middle education: 8-12 years, and higher education: >12 years), and the presence of comorbidities such as diabetes mellitus, hypertension, and dyslipidemia. Nutritional status will be classified based on BMI, which is obtained by dividing body weight by bipedal height squared (weight/height^2^), with cutoff points considered, low weight: <22.9 kg/m^2^, normal weight: 23.0 to 27.9 kg/m^2^, overweight: 28.0 to 31.9 kg/m^2^, and obesity: ≥32 kg/m^2^ [65]. The questions and the classification indicated in the National Health Survey 2016 to 2017 will be used.

### Ethical Considerations

The study was conducted in accordance with the Declaration of Helsinki [66] and approved by the University Santo Tomas Ethics Committee (CEC Accredited Res. No. 01-MZS, approved on May 24, 2024). Informed consent will be obtained from all participants involved in the study.

### Statistical Analysis

Missing data will be handled by intention-to-treat analysis (multiple imputation method) [67]. The description will be made as measures of central tendency and dispersion (continuous variables) and as percentages (categorical variables). Using the Shapiro-Wilk test, the normality of the data will be verified, and a two-step approach for transforming will be applied to the nonnormal variables [68]. Homoscedasticity will be analyzed using Levene test. A 2-way repeated measures ANOVA will be used to determine the effects of interventions. The effects of the model are the group (experimental group vs control group), the time points (before and after test), and their interaction over time (time × group). The Bonferroni post hoc test will be applied to identify statistically significant comparisons. The *t* test effect size will be determined using Cohen *d* (<0.2 insignificant; ≥0.2 and ≤0.49 small; ≥0.5 and ≤0.79 moderate; ≥0.8 large) [69]. All analyses will be performed using SPSS (version 26; IBM Corp), with a significance level of *P*<.05, and GraphPad Prism (version 8).

## Results

Recruitment will be conducted from April 1 to May 31, 2025. The 12-week intervention period will run from June 1 to August 31, 2025. Final follow-up assessments will be completed by September 15, 2025. Data analysis is scheduled from October 1 to December 31, 2025. Manuscript drafting and submission are planned for March 2026.

## Discussion

### Anticipated Findings

It is plausible that both an STR program and a face-to-face physiotherapy program have positive effects on the primary and secondary outcomes of the study. This hypothesis is based on evidence from systematic reviews and meta-analyses that supports how exercise-based interventions generate benefits in muscle strength, balance, cardiorespiratory fitness, quality of life, and the number of daily steps in older people [35,38,70,71].

### Strengths and Limitations of This Study

We expect the STR program to offer results equal to or superior to those of the face-to-face physical exercise intervention, as this type of intervention has been described as an effective strategy to overcome the commonly reported barriers to access and adherence to physical exercise [24,27,29,30]. In addition, it would be of great interest to conduct a multicenter study in the future involving different cities and countries, thus confirming whether the results expected in this study could be replicated in other latitudes and social and geographical contexts. However, factors such as self-efficacy in using technological devices, motivation to practice physical activity [72], and the perception of loss of social support by not attending group sessions with people who share similar conditions [73] could interfere with the expected results of the telerehabilitation program in the intervened older adults. However, the personalized design of the program, with direct attention from a professional in charge of delivering the sessions, is expected to mitigate these limitations effectively.

While asynchronous telerehabilitation may represent a viable alternative that overcomes many of the barriers associated with in-person therapy, future clinical trials should directly compare synchronous and asynchronous modalities to determine their respective advantages and limitations. Furthermore, it is recommended that digital literacy measurement be considered, either as an inclusion criterion or as a covariate, to better understand its effect on treatment adherence and outcomes.

### Conclusions

Implementing STR programs could represent a viable and effective alternative to in-person physical therapy for older adults who are frail. This innovative approach would not only achieve comparable or superior improvements in strength, cardiorespiratory fitness, and other functional indicators but also facilitate access to rehabilitation by overcoming geographical and logistical barriers. Telerehabilitation would allow for the personalization and optimization of therapeutic resources, improving the quality of life and independence of a vulnerable population. These potential findings would have important implications for integrating remote strategies into routine practice and for fostering future research that expands their application in clinical settings.

## Appendix

Multimedia Appendix 1 SPIRIT checklist.

Multimedia Appendix 2 Peer review report by National Agency for Research and Development (ANID), FONDECYT, Ministry of Science Technology, Knowledge and Innovation (Chile).

## Abbreviations

CIAM: Centro Integral del Adulto Mayor (Comprehensive Center for the Older Adults)
CONSORT: Consolidated Standards of Reporting Trials
FPT: face-to-face physical therapy
HRmax: maximum heart rate
RPE: Rating of Perceived Exertion
SPIRIT: Standard Protocol Items: Recommendations for Interventional Trials
STR: synchronous telerehabilitation
