# Supplementary material for: Effectiveness of Synchronous Telerehabilitation Versus Face-to-Face Physical Therapy in Older Adults Who Are Frail: Protocol for a Randomized Controlled Trial
Source: JMIR Res Protoc. 2025 Sep 16;14:e72318. doi: 10.2196/72318 (PMC12485262; doi:10.2196/72318)
Supplement: Multimedia Appendix 2 [file resprot_v14i1e72318_app2.pdf]

Machine Translated by Google

Agencia
Nacional de
Investigación
y Desarrollo

Ministerio de Ciencia,
Tecnología, Conocimiento
e Innovación

ANID/Projects Subdirectorate/No. 80

Santiago, February 1, 2024 Ref.:

Project No. 11240532

Dear Sir, IGOR CIGARROA:

On behalf of the National Research and Development Agency, I am writing to inform you that your project No. 11240532, submitted for the 2024 Fondecyt Research Initiation Competition in the MEDICINE Evaluation Group G2-G3 - CLINICS AND PUBLIC HEALTH CS, has been approved.

In this call, 1,400 projects were submitted for tender, of which 420 (30.0%) were awarded.

In the MEDICINE Group G2-G3 - CLINICS AND PUBLIC HEALTH CS. 64 proposals were entered into the competition and 19 (29.7%) were awarded. His project obtained 17th place and a score of 3.675.

Following this letter, you will be informed of the grades and comments your project received and a certificate accrediting the award of your project is included. In addition, you will have access to the following in the Online Evaluation System:

a. Approved budget for each year of execution, being able to redistribute the funds assigned annually, if necessary.
pertinent estimate.
b. Authorizations-Certifications Report, if applicable. Indicates the documents that must be submitted to start the project, within a period of three months from the date of this letter (section 11.2.1. literal d) of the contest rules). c. "Decision to Execute" button. When pressed, you must communicate
whether you accept or reject the award of the project.
a maximum period of 10 administrative business days, from the date of this letter.

Regarding the process of Signing the Agreement and Transferring resources, review the instructions available at InstructivoFirmaConvenioIniciacion2024.pdf. and consider that, in case of queries or requests related to the execution of your project, these must be sent through ANID Help (https://ayuda.anid.cl).

Please accept our congratulations for this important achievement in your scientific career.

Kind regards to you,

CAMILO ERAZO LEIVA
Deputy Director(s)
Subdirectorate of Research Projects
National Agency for Research and Development

Machine Translated by Google

Agencia Nacional de Investigación y Desarrollo
Ministerio de Ciencia, Tecnología, Conocimiento e Innovación

RESULT OF THE EVALUATION PROCESS

QUALIFICATIONS OBTAINED BY PROJECT N° 11240532

Stage 1: Quality, Feasibility and Novelty Scientific or Technological Proposal (75%)	Stage 2: Academic and professional career Research of the Applicant (25%)
3.4	4.5

EVALUATION COMMENTS
PROJECT No. 11240532

Use:

The comments issued by the Evaluation Group may highlight aspects that it considers necessary to convey to the applicants, in accordance with section 9.6 of the competition rules.

1. QUALITY, FEASIBILITY AND SCIENTIFIC OR TECHNOLOGICAL NOVELTY OF THE PROPOSAL

This project seeks to become a promising solution to resolve the barriers to physical activity practice in frail elderly people through the incorporation of a synchronous telerehabilitation program.

Theoretical framework.

The proposal is generally well written and easy to read. It addresses a relevant public health problem.
The theoretical framework clearly explains the state of the art regarding the frailty of older people, its relevance to health, and the effects of multicomponent training as a way of preventing/treating the frailty of older people.

Reference is also made to synchronous telerehabilitation as a possible therapeutic alternative to in-person rehabilitation. The IR justifies that in Chile there are few studies using mobile technologies, but in the rest of the world this is a fairly studied therapeutic proposal. This, which was little developed in the conceptual framework, is essential to establish what the knowledge gap is that will be addressed with the proposal.

Below are some recent publications by international and national authors (Eur Geriatr Med 13, 1177–1185 (2022). https://doi.org/10.1007/s41999-022-00672-y Journal of Telemedicine and Telecare DOI: 10.1177/1357633X211073256 Sanitario Vol. 21 No. 2 (2022) / DOI: https://doi.org/10.19136/hs.a21n2.4863 Brain Sci. 2020, 10(11), 773; https://doi.org/10.3390/brainsci10110773). Thus, the justification of the study seems to fundamentally adjust to the national reality.

Machine Translated by Google

Methodology

Regarding the statement of the research problem, it is easily identified.

There is a relationship between the questions, hypotheses and objectives. However, the objectives could be worded in a better way, being more direct and using more consistent terminology (name of the groups).

Specific objective SO1 is a necessary activity and is implicit in SO2.

Furthermore, the way the Hi2 hypothesis is presented is confusing with respect to the approach to the RCT (is an effect sought that is equal or greater? or just greater? This modifies the statistical plan). There is also the question of how SO3 will be fulfilled with the proposed methodology. It is very ambitious and is not specified later in the methodology (The concept of "clinical significance" is not addressed in the conceptual framework).

The inclusion and exclusion criteria are clear and consistent with the study. The details of the randomization are set out and both the interventions and the measurements are well developed. More innovative measurements could be incorporated with the intention of associating with the mechanisms by which the expected effectiveness is greater in one protocol than in the other.

It seems that one of the groups is considering institutionalized elderly people and another group will be at home, but the description of the first group is not very clear in the proposal.

The study includes a vulnerable group that is not required to be digitally literate to carry out STR. This could modify the results. It would be advisable to consider at least sufficient training to enable older people (it is estimated that these are simple instructions, but we do not know the opinion of older people regarding this strategy). Nor is it included personnel who support during telerehabilitation (considering the feasibility of the therapy).

Given the characteristics of the proposal, it would seem better to consider an approach as a "Non-inferiority Study", rather than a superiority study. This is considering that for the calculation of the sample size, an effect size of 0.80 was assumed, for which sufficient arguments are not provided; it is not about comparing an intervention versus a control management without intervention, but rather two different interventions.

Furthermore, it is not possible to follow a possible superiority of the telerehabilitation option from the arguments of IR. Nor is it described whether the study will have an intention-to-treat approach (there are potential implementation problems for both STR and FPT).

The repeated measures ANOVA test is useful for assessing the effect size of interventions (within group), but the strategy that will be used to compare interventions is not explicit.

Given the potential accessory difficulties in subgroups of frail older people, it would be appropriate to stratify not only by sex, but also by other characteristics such as age, hearing, speech difficulties, etc.
vision.

(All of the above are cardinal points, because the feasibility of the study is derived from them)

The work plan is coherent, but what is striking is that according to the Gantt chart all participants will be enrolled in the same 3 months, which seems quite unlikely, but rather they will enter over the course of 3 years.

Machine Translated by Google

likely.

Overall, the project is considered "Good", with methodological flaws to overcome.

2. ACADEMIC AND RESEARCH CAREER OF THE APPLICANT

For the evaluation of this item, the information presented by the responsible researcher (IR) was considered with the background duly accredited in the Annexes section.

The IR presents a story in his biographical review and career that allows us to evaluate his good capabilities to develop the proposal.

The IR is a kinesiologist with a Master's degree and subsequent PhD in Neurosciences from the Autonomous University of Barcelona.

The IR has obtained scholarships from CONICYT for his PhD and from Santander Research for one of his Postdocs carried out at the University of Granada. The other Postdoc was carried out at the University of Elche. His CV also includes the award of FONDEPORTE, his participation as co-investigator in an international project and his academic position as Associate Professor at the Universidad Santo Tomás.

The IR has contributed in this direction from a scientific and educational perspective, exploring the subject and training undergraduate and graduate students from our country.

The scientific productivity of the IR has been prolific with 90 manuscripts published since 2018. In the selection, it identifies 9 articles and 1 book chapter in which it appears as first author.

In the presentation of his biographical sketch and career, several activities of fundamentally academic extension are identified, including participation in international research networks and scientific societies. Reference is made to an activity of linkage with the society/ community, without prejudice to the relevance and potential impact of his line of research in this dimension. Thus, a greater link with the non-specialized community/society where he can disseminate knowledge is desirable.

Machine Translated by Google

Agencia Nacional de Investigación y Desarrollo
Ministerio de Ciencia, Tecnología, Conocimiento e Innovación

CERTIFICATE OF AWARD

FONDECYT COMPETITION FOR INTRODUCTION TO RESEARCH

February 1, 2024

Camilo Erazo Leiva, Deputy Director (s), Subdirectorate of Research Projects, of the National Agency for Research and Development, certifies that Mr. IGOR IVAN CIGARROA CUEVAS, has awarded the project N°11240532 in the FONDECYT Research Initiation Project Competition 2024, entitled EFFECTIVENESS OF SYNCHRONOUS TELEREHABILITATION VERSUS FACE-TO-FACE PHYSICAL THERAPY ON PHYSICAL FITNESS, FUNCTIONAL STATUS, AND QUALITY OF LIFE IN FRAIL OLDER ADULTS.

The project, sponsored by the SANTO TOMAS UNIVERSITY, contemplates a duration of 3 years – from 15 –, and financing of $34,266,000, from March 2024 to March 14, 2027 $30,420,000 and $21,840,000, for each year of execution, respectively.

This certificate is issued to the interested party for the purposes he/she deems appropriate.

CAMILO ERAZO LEIVA
Deputy Director(s)
Subdirectorate of Research Projects
National Agency for Research and Development

Moneda nº1375,
Santiago, Chile
Tel: 56 (2) 2365 4600
www.anid.cl
@AnidInforma

Gobierno de Chile
